# Predictors of rituximab efficacy in systemic sclerosis-associated interstitial lung disease: machine-learning analysis of the DESIRES trial

**DOI:** 10.1093/rheumatology/keae716

**Published:** 2024-12-24

**Authors:** Ai Kuzumi, Koji Oba, Satoshi Ebata, Kosuke Kashiwabara, Keiko Ueda, Yukari Uemura, Takeyuki Watadani, Takemichi Fukasawa, Shunsuke Miura, Asako Yoshizaki-Ogawa, Hidenori Kage, Shinichi Sato, Ayumi Yoshizaki

**Affiliations:** Department of Dermatology, Graduate School of Medicine, The University of Tokyo, Tokyo, Japan; Department of Biostatistics, School of Public Health, The University of Tokyo, Tokyo, Japan; Department of Dermatology, Graduate School of Medicine, The University of Tokyo, Tokyo, Japan; Clinical Research Support Center, Tokyo University Hospital, Tokyo, Japan; Clinical Research Support Center, Tokyo University Hospital, Tokyo, Japan; Clinical Research Support Center, Tokyo University Hospital, Tokyo, Japan; Biostatistics Section, Department of Data Science, Center for Clinical Sciences, National Center for Global Health and Medicine, Tokyo, Japan; Department of Diagnostic Radiology, Graduate School of Medicine, The University of Tokyo, Tokyo, Japan; Department of Dermatology, Graduate School of Medicine, The University of Tokyo, Tokyo, Japan; Department of Clinical Cannabinoid Research, Graduate School of Medicine, The University of Tokyo, Tokyo, Japan; Department of Dermatology, Graduate School of Medicine, The University of Tokyo, Tokyo, Japan; Department of Dermatology, Graduate School of Medicine, The University of Tokyo, Tokyo, Japan; Department of Respiratory Medicine, Graduate School of Medicine, The University of Tokyo, Tokyo, Japan; Department of Dermatology, Graduate School of Medicine, The University of Tokyo, Tokyo, Japan; Department of Dermatology, Graduate School of Medicine, The University of Tokyo, Tokyo, Japan; Department of Clinical Cannabinoid Research, Graduate School of Medicine, The University of Tokyo, Tokyo, Japan

**Keywords:** systemic sclerosis, interstitial lung disease, rituximab, machine learning

## Abstract

**Objectives:**

Rituximab is emerging as a promising therapeutic option for systemic sclerosis-associated interstitial lung disease (SSc-ILD). However, little is known about factors that predict the efficacy of rituximab in SSc-ILD.

**Methods:**

A post-hoc analysis was performed on prospective data from 48 patients with SSc-ILD in the double-blind, randomized, placebo-controlled DESIRES trial. A total of 28 baseline factors were selected as candidates to predict the efficacy of rituximab on the percentage of predicted forced vital capacity (ppFVC) at 24 weeks. A machine learning causal tree algorithm was used to explore the combination of predictors to identify subpopulations with a good response to rituximab.

**Results:**

Serum levels of C-reactive protein (CRP) and Krebs von den Lungen-6 (KL-6) were selected as branches of the decision tree to stratify patients into three subpopulations. In the subpopulation with serum CRP levels ≥0.055 mg/dl, ΔppFVC was significantly higher in the rituximab group than in the placebo group [difference 8.01% (95% CI: 4.40%, 11.62%)]. In the subpopulation with serum CRP levels <0.055 mg/dl and serum KL-6 levels ≥364 U/ml, ΔppFVC was comparable between the two groups [difference 2.47% (95% CI: −1.99%, 6.92%)]. In the subpopulation with serum CRP levels <0.055 mg/dl and serum KL-6 levels <364 U/ml, ΔppFVC was significantly lower in rituximab than in placebo [difference −6.85% (95% CI: −10.80%, −2.91%)].

**Conclusion:**

Even slight elevations in serum CRP levels are associated with the improvement in ppFVC and may serve as predictors of rituximab efficacy in SSc-ILD.

Rheumatology key messages

- Serum levels of CRP and KL-6 may serve as predictors of rituximab efficacy in SSc-ILD.
- Slight elevations in serum CRP levels may indicate a good response to rituximab in SSc-ILD.

## Introduction

Systemic sclerosis (SSc) is a connective tissue disease characterized by excessive fibrosis in the skin and internal organs, such as the lungs, heart and kidneys [1]. It has the worst prognosis among connective tissue diseases, with a 10-year mortality rate of about 30% [2]. In particular, interstitial lung disease (ILD) affects 35–50% of patients and is the leading cause of death in SSc [3]. Despite the high disease burden, an optimal treatment for SSc-ILD has not yet been established.

Although the pathogenesis of SSc remains elusive, increasing evidence suggests that B cells play a key role in the development of the disease [4, 5]. In line with this, B-cell depletion with rituximab, a chimeric monoclonal antibody against human CD20, is emerging as a potential treatment for SSc [6]. Recently, we have demonstrated the efficacy of rituximab for skin sclerosis in SSc in the double-blind, investigator-initiated, randomized, placebo-controlled DESIRES trial [7, 8]. In this study, the secondary end point of the percentage of predicted forced vital capacity (ppFVC) also improved significantly in the rituximab group compared with placebo, supporting the efficacy of rituximab in SSc-ILD. However, there is considerable heterogeneity in the improvement of ppFVC with rituximab [9, 10] and little is known about prognostic factors that predict response to rituximab in SSc-ILD.

Recent advances in machine learning now allow us to explore factors that predict progression, prognosis and treatment response in a post-hoc data analysis [11–13]. Using a machine learning classification and causal tree algorithm [14], we have previously identified predictors of rituximab efficacy in skin sclerosis from the DESIRES trial data [15]. Here, we used the same approach to investigate predictors of rituximab efficacy in SSc-ILD.

## Methods

### Study design

This study enrolled patients with SSc-ILD who participated in the DESIRES trial (NCT04274257) and received at least one dose of rituximab or placebo. The study design of the DESIRES trial has been previously reported [7, 8]. Briefly, the trial consisted of two phases: a randomized, double-blind, placebo-controlled phase of 24 weeks and a subsequent open-label extension phase of 24 weeks. The inclusion criteria were (i) age of 20–79 years; (ii) meeting the 2013 American College of Rheumatology and European League Against Rheumatism classification criteria for SSc [16]; (iii) modified Rodnan skin score (MRSS) of 10 or greater [17]; (iv) not receiving corticosteroids equivalent to >10 mg/day of prednisolone within 2 weeks prior to study treatment; and (5) not receiving imatinib, immunosuppressants, anti-fibrotic agents, high-dose intravenous immunoglobulin or other investigational products within 4 weeks prior to study treatment.

### Patients

In the DESIRES trial, 56 patients were enrolled and assigned to treatment arms, with 54 patients receiving at least one dose of rituximab or placebo. Of these patients, 48 patients were diagnosed with SSc-ILD and therefore included in this study. The presence or absence of SSc-ILD was determined by an experienced radiologist (T.W.) based on high-resolution CT images. The extent of the ILD lesion was quantitatively assessed using OsiriX v12 image analysis software (Pixmeo SARL, Bernex, Switzerland). This study was approved by the ethics committee of the University of Tokyo Graduate School of Medicine and conducted in accordance with the Declaration of Helsinki. All patients provided written informed consent.

### Outcome

The primary end point was the absolute change in ppFVC (ΔppFVC) at 24 weeks compared with the baseline. Respiratory function tests were performed according to international guidelines. Missing data were replaced with the last previous observation for three patients (all for placebo) who did not have a measurement of ppFVC at 24 weeks.

### Candidates for baseline predictors

We searched the PubMed and MEDLINE databases for review articles on biomarkers of SSc that were published in the past 15 years. Based on the description of the review articles [18–20] and discussion among members of the Japan SSc Study Group, the following 28 variables at baseline were selected as candidate prognostic factors: eight categorical variables (sex, previous use of immunosuppressants and/or biologics, diffuse cutaneous SSc [21], puffy fingers, skin ulcer, reflux esophagitis, Barret’s esophagus and arthritis) and 20 continuous variables [age, disease duration, MRSS, titres of anti-topoisomerase I, anti-centromere, and anti-RNA polymerase III antibodies, number of CD19-positive cells and CD20-positive cells in peripheral blood, ppFVC, diffusing capacity for carbon monoxide, serum levels of Krebs von den Lungen-6 (KL-6), surfactant protein A, surfactant protein D, total protein, C-reactive protein (CRP), IgG, IgM, brain natriuretic peptide, and haemoglobin, and area occupied by interstitial shadows of lung fields on high-resolution CT images]. Serum CRP levels were measured by the conventional CRP assays.

### Data and statistical analysis

A data-driven approach proposed by Athey and Imbens [14] was used to partition the data into subpopulations that showed heterogeneity in the treatment effect of rituximab. The algorithm used is called a causal tree, which incorporates many similar ideas to those related to classification and regression trees [22] but focuses on estimating heterogeneous treatment effects. First, the tree is grown from the root node using an honest version of the splitting rule that is based on the expected mean squared error. At each node, the data in a leaf was split into two groups to best minimize the risk function. The splitting routine was applied recursively until the routine stopped if it could not produce splits with at least five patients from the rituximab and placebo groups in each terminal node. Next, we used 5-fold cross-validation to prune the tree to minimize the normalized cross-validation error. The analysis was performed in R version 4.0.3 with the causalTree package [23]. The Wilcoxon rank sum test or Fisher’s exact test was used to compare baseline predictors between rituximab and placebo. A linear trend test or a Mantel trend test was used to compare baseline predictors between subpopulations that are based on the causalTree algorithm. The Pearson correlation coefficient was used to determine the relationship between two variables in a scatter plot. Other analyses were performed using SAS ver. 9.4 and JMP PRO 16.0 (SAS Institute Inc., Cary, NC, USA).

## Results

### Patients

Of 48 patients in this study, 25 received rituximab and the remaining 23 received placebo in the double-blind phase of the DESIRES trial. Baseline characteristics were comparable between the two groups, including 28 candidates for baseline predictors (Table 1).

**Table 1.** Demographic and clinical characteristics of the study population

Characteristic	Total (*n* = 48)	Placebo (*n* = 23)	Rituximab (*n* = 25)	*P* value
Female	44 (91.7%)	21 (91.3%)	23 (92.0%)	1.000
Age, years	48.0 (27–78)	48.0 (27–66)	47.0 (27–78)	1.000
Disease duration, months^a^	76.5 (5–341)	57.0 (9–248)	80.0 (5–341)	0.317
MRSS	13.5 (10–28)	14.0 (10–28)	13.0 (10–21)	0.434
Previous use of immunosuppressants and biologics	26 (54.2%)	15 (65.2%)	11 (44.0%)	0.161
Diffuse cutaneous systemic sclerosis	39 (81.3%)	19 (80.0%)	20 (82.6%)	1.000
Puffy fingers	41 (85.4%)	19 (82.6%)	22 (88.0%)	0.696
Skin ulcer	32 (66.7%)	16 (69.6%)	16 (64.0%)	0.765
Reflux esophagitis	44 (91.7%)	21 (91.3%)	23 (92.0%)	1.000
Barret esophagus	7 (14.6%)	3 (13.0%)	4 (16.0%)	1.000
Arthritis	13 (27.1%)	5 (21.7%)	8 (32.0%)	0.523
Anti-topoisomerase I antibody positive	28 (58.3%)	13 (56.5%)	15 (60.0%)	1.000
Anti-topoisomerase I antibody titre, U/ml	93.5 (0–182)	106.0 (0–182)	92.2 (1–176)	0.983
Anti-centromere antibody positive	5 (10.4%)	2 (8.7%)	3 (12.0%)	1.000
Anti-centromere antibody titre, U/ml	4.9 (1.9–184.0)	4.9 (1.9–164.0)	4.9 (1.9–184.0)	0.498
RNA polymerase III antibody positive	7 (14.6%)	2 (8.7%)	5 (20.0%)	0.419
RNA polymerase III antibody titre, U/ml	4.9 (4.9–145.0)	4.9 (4.9–145.0)	4.9 (4.9–131.0)	0.308
CD19-positive cells, cells/µl	180.0 (16–579)	191.0 (16–579)	159.0 (26–456)	0.720
CD20-positive cells, cells/µl	180.0 (15–569)	186.0 (15–569)	168.0 (37–466)	0.559
Percent-predicted FVC, %	85.8 (63.2–159.6)	85.2 (70.8–159.6)	87.7 (63.2–123.8)	0.984
Percent-predicted DLco, %	79.5 (38.7–125.4)	80.1 (38.7–109.7)	78.9 (42.1–125.4)	0.790
Serum SP-A levels, ng/ml	33.1 (13.2–148.7)	37.7 (20.8–148.7)	31.2 (13.2–118.9)	0.391
Serum SP-D levels, ng/ml	141.4 (25.5–661.0)	147.5 (29.3–661.0)	132.0 (25.5–362.5)	0.743
Serum KL-6 levels, ng/ml	453.5 (95.0–4598.0)	488.0 (179.0–4598.0)	428.0 (95.0–2534.0)	0.525
Area occupied with interstitial shadows, % of lung	11.0 (1–58)	11.0 (1–58)	11.0 (1–52)	0.659
Serum CRP levels, mg/l	0.0 (0.0–1.9)	0.1 (0.0–1.9)	0.0 (0.0–1.2)	0.196
Serum IgG levels, mg/l	1305.0 (703–2909)	1270.0 (766–2909)	1308.0 (703–2214)	0.918
Serum IgM levels, mg/l	114.5 (18–222)	100.0 (18–174)	222.0 (45–222)	0.103
Haemoglobin levels, g/dl	12.5 (8.9–14.4)	12.0 (8.9–14.2)	12.8 (10.0–14.4)	0.103
Total serum protein levels, g/dl	7.0 (5.6–8.2)	7.0 (5.6–8.2)	7.0 (5.9–7.9)	0.744
Serum BNP levels, pg/ml	17.4 (4.0–274.6)	18.8 (4.0–58.2)	14.4 (4.0–274.6)	0.141

Data are n (%) or median (range).

a Months since the onset of the first symptoms other than Raynaud’s phenomenon. BNP: brain natriuretic peptide; CRP: C-reactive protein; DL_CO_: diffusion capacity of the lungs for carbon monoxide; FVC: forced vital capacity; KL-6: Krebs von den Lungen-6; MRSS: modified Rodnan skin score; SP-A: surfactant protein-A; SP-D: surfactant protein-D.

### Subpopulations with different effect of rituximab on ppFVC

**Figure 1.**
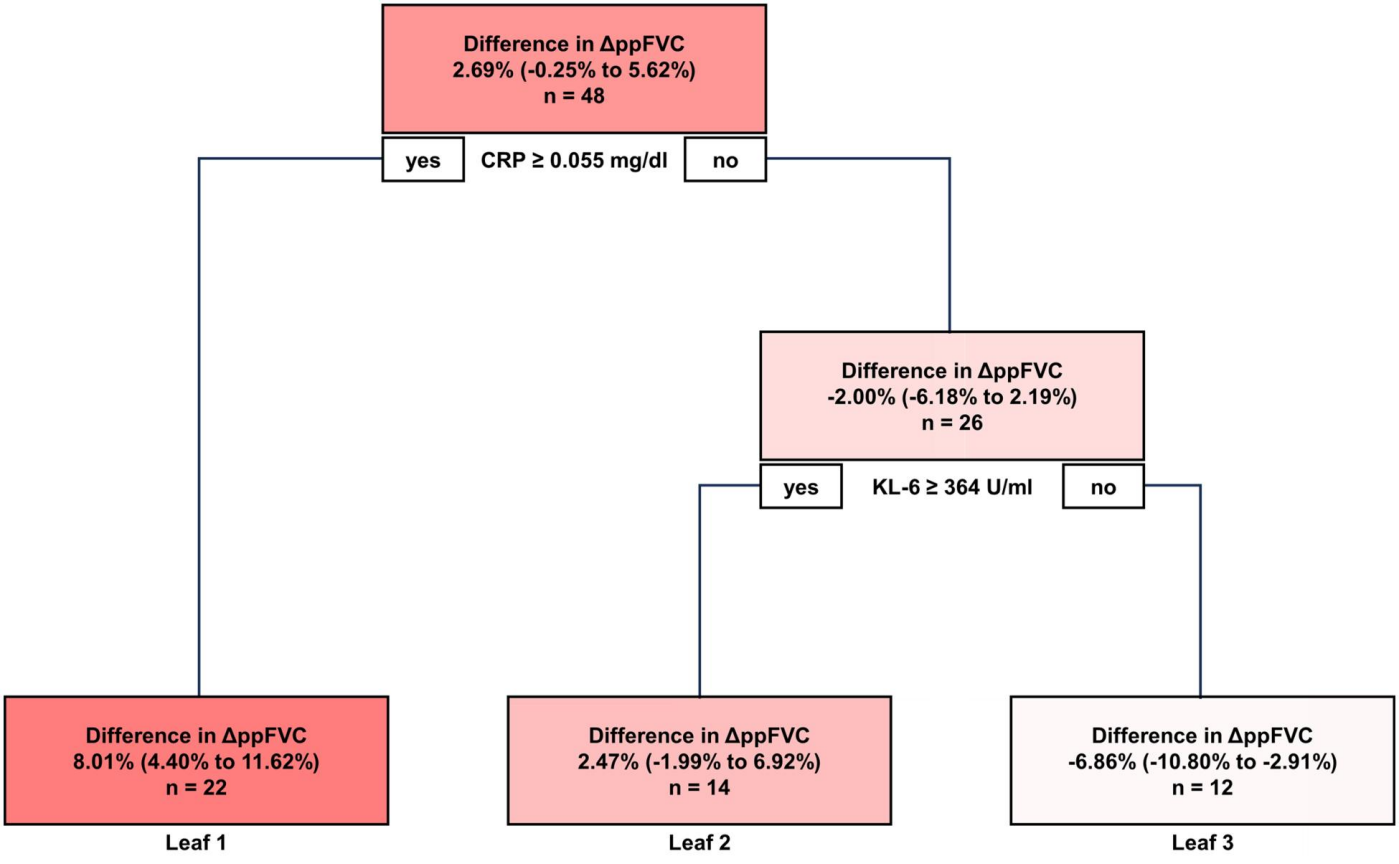
Decision tree of predictors of the effect of rituximab on ppFVC. The causal tree algorithm divided the patients into three subpopulations with different effect of rituximab on ppFVC in the DESIRES trial. The boxes show the mean difference in ΔppFVC from baseline to 24 weeks between the rituximab and placebo groups [ΔppFVC (rituximab) – ΔppFVC (placebo)] with 95% CI in parentheses. The number of patients is also shown for each subpopulation. CRP: C-reactive protein; KL-6: Krebs von den Lungen-6

The causal tree algorithm divided the patients into three subpopulations with different effect of rituximab on ΔppFVC at 24 weeks (Fig. 1). The first branch of the tree was selected for serum CRP levels; the subpopulation of patients with serum CRP levels ≥0.055 mg/dl was defined as Leaf 1 and represented 46% (*n* = 22) of all patients. For patients with serum CRP levels <0.055 mg/dl, serum KL-6 levels were selected as the next branch; the subpopulation of patients with KL-6 levels ≥364 U/ml and serum CRP levels <0.055 mg/dl was defined as Leaf 2, and the subpopulation of patients with serum CRP levels <0.055 mg/dl and KL-6 levels <364 U/ml was defined as Leaf 3. Leaves 2 and 3 represented 29% (*n* = 14) and 25% (*n* = 12) of all patients, respectively. In Leaf 1, 77% of the patients [17/22 (7/10 in rituximab and 10/12 in placebo)] presented with serum KL-6 levels ≥364 U/ml.

The baseline characteristics in each subpopulation are shown in Table 2. Among the leaves, a statistically significant linear trend was observed in distributions of selected predictors (serum CRP and KL-6 levels) and several other factors (ppFVC, diffusing capacity for carbon monoxide, area occupied with interstitial shadows of lung fields, and serum SP-A, IgG and total protein levels).

**Table 2.** Demographic and clinical characteristics of subpopulations

Characteristic	Leaf 1 (*n* = 22)	Leaf 2 (*n* = 14)	Leaf 3 (*n* = 12)	*P* value for trend test
Female	20 (90.9%)	14 (100.0%)	10 (83.3%)	0.597
Age, years	47.5 (27–71)	49.0 (35–78)	45.0 (27–66)	0.214
Disease duration, months^a^	84.5 (5–248)	59.5 (12–341)	58.5 (5–193)	0.530
MRSS	13.0 (10–28)	13.5 (10–20)	14.0 (10–24)	0.971
Previous use of immunosuppressants and biologics	16 (72.7%)	4 (28.6%)	6 (50.0%)	0.107
Diffuse cutaneous systemic sclerosis	17 (77.3%)	12 (85.7%)	10 (83.3%)	0.614
Puffy fingers	19 (86.4%)	12 (85.7%)	10 (83.3%)	0.820
Skin ulcer	13 (59.1%)	9 (64.3%)	10 (83.3%)	0.173
Reflux esophagitis	21 (95.5%)	12 (85.7%)	11 (91.7%)	0.597
Barret esophagus	5 (22.7%)	2 (14.3%)	0 (0.0%)	0.079
Arthritis	7 (31.8%)	2 (14.3%)	4 (33.3%)	0.909
Anti-topoisomerase I antibody positive	13 (59.1%)	9 (64.3%)	6 (50.0%)	0.679
Anti-topoisomerase I antibody titre, U/ml	100.4 (1.0–161.0)	103.1 (1.0–182.0)	37.4 (0.0–159.0)	0.655
Anti-centromere antibody positive	3 (13.6%)	0 (0.0%)	2 (16.7%)	0.981
Anti-centromere antibody titre, U/ml	4.9 (1.9–164.0)	4.9 (1.9–4.9)	4.9 (1.9–184.0)	0.829
RNA polymerase III antibody positive	3 (13.6%)	2 (14.3%)	2 (16.7%)	0.820
RNA polymerase III antibody titre, U/ml	4.9 (4.9–131.0)	4.9 (4.9–115.0)	4.9 (4.9–145.0)	0.542
CD19-positive cells, cells/µl	194.0 (26–579)	175.0 (23–450)	153.0 (16–537)	0.242
CD20-positive cells, cells/µl	194.0 (37–569)	175.5 (22–464)	157.0 (15–532)	0.220
Percent-predicted FVC, %	83.7 (64.3–109.8)	87.0 (63.2–123.8)	94.3 (68.6–159.6)	0.033*
Percent-predicted DLco, %	75.4 (38.7–98.9)	78.9 (60.8–122.4)	100.6 (70.1–125.4)	<0.001***
Serum SP-A levels, ng/ml	39.0 (13.3–148.7)	35.1 (29.0–92.5)	27.4 (13.2–48.4)	0.039*
Serum SP-D levels, ng/ml	150.1 (25.5–362.5)	545.0 (381.0–2534.0)	196.0 (95.0–347.0)	0.129
Serum KL-6 levels, ng/ml	698.0 (158.0–4598.0)	545.0 (381.0–2534.0)	196.0 (95.0–347.0)	0.005**
Area occupied with interstitial shadows, % of lung	13.5 (2–58)	13.0 (4–38)	2.5 (1–14)	0.003**
Serum CRP levels, mg/l	0.2 (0.1–1.9)	0.0 (0.0–0.0)	0.0 (0.0–0.1)	0.001**
Serum IgG levels, mg/l	1496.0 (766–2909)	1263.0 (942–2214)	1179.5 (703–1692)	0.037*
Serum IgM levels, mg/l	120.0 (32–189)	100.5 (47–208)	112.0 (18–222)	0.906
Haemoglobin levels, g/dl	12.2 (9.9–14.2)	12.9 (10.0–14.4)	12.4 (8.9–13.8)	0.666
Total serum protein levels, g/dl	7.3 (6.4–8.2)	6.9 (5.9–7.9)	6.9 (5.6–7.5)	0.007**
Serum BNP levels, pg/ml	15.4 (4.0–274.6)	17.8 (4.0–31.8)	19.3 (7.3–55.4)	0.355

Data are *n* (%) or median (range).

a Months since the onset of the first symptoms other than Raynaud’s phenomenon.

* *P* < 0.05,

** *P* < 0.01 and

*** *P* < 0.001.

BNP: brain natriuretic peptide; CRP: C-reactive protein; DL_CO_: diffusion capacity of the lungs for carbon monoxide; FVC: forced vital capacity; KL-6: Krebs von den Lungen-6; MRSS: modified Rodnan skin score; SP-A: surfactant protein-A; SP-D: surfactant protein-D.

### The difference in ΔppFVC between rituximab and placebo in each subpopulation

**Figure 2.**
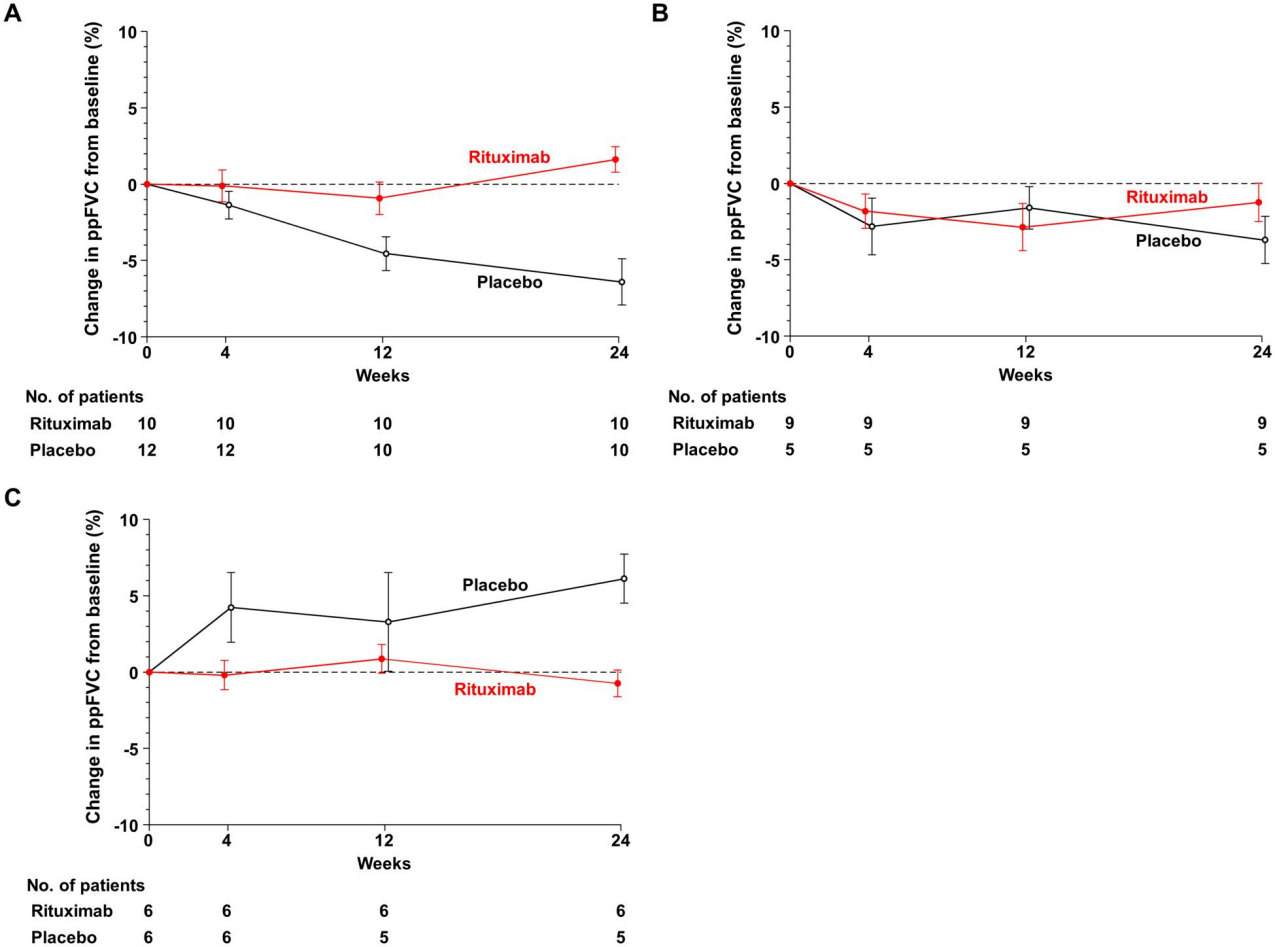
Change in ppFVC from baseline to 24 weeks in each subpopulation. The mean ΔppFVC from baseline to 24 weeks in the placebo and rituximab groups in the following three subpopulations derived by the causal tree algorithm; (**A**) CRP levels ≥0.055 mg/dl (Leaf 1), (**B**) serum CRP levels <0.055 mg/dl and serum KL-6 levels ≥364 U/ml (Leaf 2), and (**C**) serum CRP levels <0.055 mg/dl and serum KL-6 levels <364 U/ml (Leaf 3). Bars indicate the 95% CIs. CRP: C-reactive protein; FVC: forced vital capacity; KL-6: Krebs von den Lungen-6

Next, we compared ΔppFVC between rituximab and placebo during the double-blind and open-label phases in each subpopulation. Leaf 1 with serum CRP levels ≥0.055 mg/dl showed the greatest improvement in ppFVC in the rituximab group and was the only subpopulation where ΔppFVC was significantly higher in the rituximab group than in the placebo group at week 24 [ΔppFVC 1.61% *vs* −6.40%; difference 8.01% (95% CI: 4.40%, 11.62%), *P* =0.0002; Fig. 2A]. In the open-label phase, in which all patients received rituximab, the difference in ΔppFVC between the two groups was largely maintained, while ppFVC improved in both the rituximab-rituximab and placebo-rituximab groups [ΔppFVC 2.49% *vs* −7.04%; difference 9.53% (95% CI: 5.99%, 13.06%), *P* <0.0001 at 36 weeks; ΔppFVC 3.31% *vs* −2.50%; difference 5.81% (95% CI: −0.91%, 12.53%), *P* =0.085 at 48 weeks; Supplementary Fig. S1A, available at *Rheumatology* online]. These results suggest that Leaf 1 represents good candidates for rituximab in SSc-ILD. In Leaf 2 with serum CRP levels <0.055 ng/ml and serum KL-6 levels ≥364 U/ml, ΔppFVC tended to be higher in rituximab than in placebo, but it did not reach a statistical significance at week 24 [ΔppFVC −1.23% *vs* −3.70%; difference 2.47% (95% CI: −1.99%, 6.92%); *P* =0.251; Fig. 2B]. In this subpopulation, ΔppFVC remained almost unchanged in both the rituximab and placebo groups during the open-label phase [ΔppFVC −1.00% *vs* −2.64%; difference 1.64% (95% CI: −5.64%, 8.92%), *P* =0.630 at 36 weeks; ΔppFVC −1.90% *vs* −3.15%; difference 1.25% (95% CI: −5.57%, 8.07%), *P* =0.692 at 48 weeks; Supplementary Fig. S1B, available at *Rheumatology* online]. In Leaf 3 with serum CRP levels <0.05 mg/dl and serum KL-6 levels <364 U/ml, ΔppFVC was significantly lower in rituximab than in placebo at week 24 [ΔppFVC −0.73% *vs* 6.12%; difference −6.86% (95% CI: −10.80%, −2.91%); *P* =0.003; Fig. 2C]. In this subpopulation, there was no decline in ppFVC in either the rituximab or placebo groups throughout the 48-week follow-up, suggesting that Leaf 3 is a group of patients at low risk of SSc-ILD progression [ΔppFVC 0.93% *vs* 6.23%; difference −5.29% (95% CI: −11.76%, 1.18%), *P* =0.096 at 36 weeks; ΔppFVC 0.46% *vs* 2.56%; difference −2.36% (95% CI: −9.01%, 1.69%), *P* =0.156 at 48 weeks; Supplementary Fig. S1C, available at *Rheumatology* online].

### Correlation between baseline predictors and ΔppFVC

**Figure 3.**
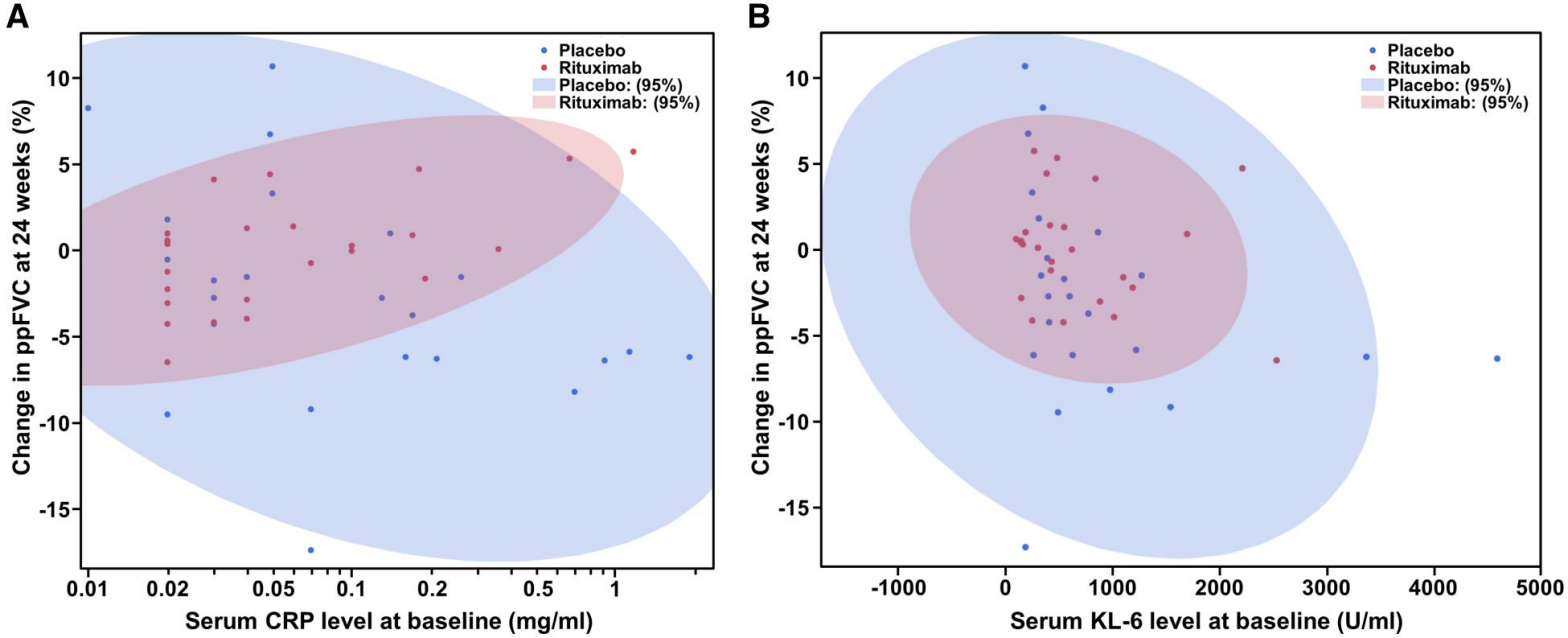
Correlations between selected baseline predictors and ΔppFVC. Correlations between baseline serum CRP (**A**) and KL-6 (**B**) levels and ΔppFVC from baseline to 24 weeks. Shaded areas indicate 95% CI regions. CRP: C-reactive protein; FVC: forced vital capacity; KL-6: Krebs von den Lungen-6

**Figure 4.**
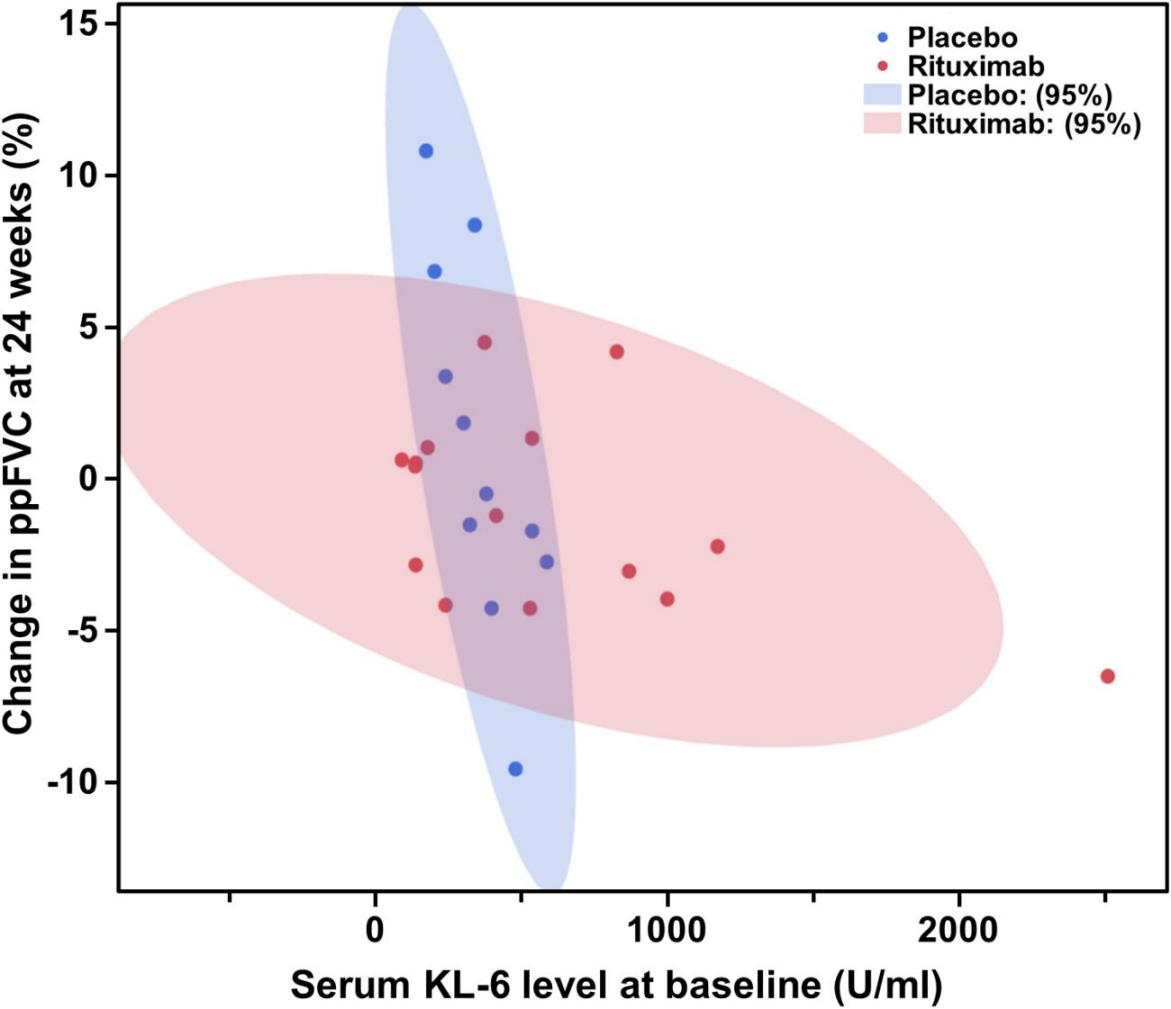
Correlations between baseline serum KL-6 levels and ΔppFVC in patients with baseline serum CRP levels <0.055 mg/dl. Correlations between baseline serum KL-6 levels and ΔppFVC from baseline to 24 weeks in patients with baseline serum CRP levels <0.055 mg/dl. Shaded areas indicate 95% CI regions. FVC: forced vital capacity; KL-6: Krebs von den Lungen-6

We subsequently analysed the correlation between serum CRP or KL-6 levels at baseline and ΔppFVC from baseline to 24 weeks in the rituximab and placebo groups. There was a moderate positive correlation between serum CRP levels and ΔppFVC in the rituximab group (r = 0.541) and a weak negative correlation in the placebo group (r = −0.253; Fig. 3A). In addition, there were slight negative correlations between serum KL-6 levels and ΔppFVC in both the rituximab and placebo groups (r = −0.188 for rituximab, r = −0.294 for placebo; Fig. 3B). We then focused on the subpopulation that split at the first branch of the decision tree by serum CRP levels <0.055 mg/dl. In this subpopulation, there was a moderate negative correlation between serum KL-6 levels and ΔppFVC in the rituximab group (r = −0.495) and a strong negative correlation in the placebo group (r = −0.768; Fig. 4), suggesting that serum KL-6 elevation indicates a good candidate for rituximab in SSc-ILD patients with serum CRP levels <0.055 mg/dl.

## Discussion

This is the first study to identify baseline factors that predict the efficacy of rituximab in SSc-ILD based on data from a randomized controlled trial. The causal tree algorithm identified serum CRP and KL-6 levels as baseline predictors of improvement in ppFVC with rituximab (Fig. 1, Table 1). The subpopulation with serum CRP levels ≥0.055 mg/dl (Leaf 1) represented the patients with the greatest improvement in ppFVC with rituximab, followed by those with serum CRP levels <0.055 mg/dl and KL-6 levels ≥364 U/ml (Leaf 2) and those with serum CRP levels <0.055 mg/dl and KL-6 levels <364 U/ml (Leaf 3). Importantly, these subpopulations showed different trajectories of ppFVC in both the rituximab and placebo groups during the 48-week follow-up period (Fig. 2).

The first branch of the decision tree was selected for serum CRP levels of 0.055 mg/dl (Fig. 1), which are considerably below the upper limit of its standard range (<0.30 mg/dl). In the subpopulation of serum CRP levels ≥0.055 mg/dl, the decrease in ppFVC in the placebo group was numerically greater than in the other subpopulations (Fig. 2), suggesting that even subclinical inflammation is associated with a decrease in ppFVC. This result not only extends previous findings of an association between serum CRP levels ≥0.5–0.6 mg/dl and progression of SSc-ILD [24, 25], but is also in line with recent studies showing that the slight elevation of serum CRP levels predict poor prognosis in various chronic diseases [26–28]. Of note, the efficacy of rituximab on ppFVC improvement was most pronounced in this subpopulation with serum CRP levels ≥0.055 mg/dl (Fig. 1). Taken together, these results suggest that rituximab is a good indication for SSc-ILD at high risk of progression with subclinical inflammation.

Serum KL-6 levels were selected for the second branch of the decision tree after serum CRP levels (Fig. 1). KL-6 is a glycoprotein produced primarily by type II pneumocytes and is widely recognized as a prognostic marker in SSc-ILD [29, 30]. Consistently, serum KL-6 levels negatively correlated with ΔppFVC in patients receiving placebo (Fig. 3B). In particular, there was a strong negative correlation between serum KL-6 levels and ΔppFVC in patients with serum CRP levels <0.055 mg/dl (Fig. 4), suggesting that serum KL-6 levels may particularly serve as a prognostic factor for SSc-ILD in the absence of subclinical inflammation. As ΔppFVC in patients with serum KL-6 levels ≥364 U/ml and serum CRP levels <0.55 mg/dl tended to be higher in the rituximab group than in the placebo group at 24 weeks without reaching significance (Fig. 2B), further studies are needed to validate the efficacy of rituximab in SSc-ILD in this subpopulation. In addition, it should be interpreted with caution that ΔppFVC was lower in the rituximab group than in the placebo group at 24 weeks in the subpopulation with serum KL-6 levels <364 U/ml and serum CRP levels <0.55 mg/dl (Fig. 2C). The reason of this paradoxical result is unclear. However, given that there was no decline in ppFVC throughout the 48-week follow-up period in either the rituximab-rituximab or placebo-rituximab groups in these patients (Supplementary Fig. S1C, available at *Rheumatology* online), this subpopulation, ‘double negative’ for CRP and KL-6, may represent patients with a poor response to rituximab but at a low risk of progression in the first place.

Limitations of this study include the small sample size. Although we restricted the number of patients in the rituximab and placebo groups in each leaf to a minimum of five and used cross-validation to ensure a more generalized model performance evaluation, some subgroup analyses had limited power to draw definite conclusions. The paradoxical result of ΔppFVC in the subpopulation with serum KL-6 levels <364 U/ml and serum CRP levels <0.55 mg/dl at 24 weeks (Fig. 2C), where ΔppFVC was significantly lower with rituximab than with placebo, should also be validated in a larger number of patients. In addition, the severity of SSc-ILD was relatively mild in our cohort. The results of this study should be validated in future studies with larger sample size and different severity of SSc-ILD. Ideally, such studies should use not only ppFVC but also other markers to assess the efficacy of rituximab in SSc-ILD. For instance, ppFVC, serum surfactant protein-D levels and the area occupied by interstitial shadows of lung fields on high-resolution CT images were significantly improved in the rituximab group compared with placebo, while the changes in serum KL-6 levels and the percentage of predicted diffusing capacity for carbon monoxide were comparable between the two groups at 24 weeks in patients with SSc-ILD in the DESIRES trial [7]. Although ppFVC has been associated with prognosis in SSc-ILD [31–33], the use of multiple markers, including clinical, physiological and serological ones may provide a more comprehensive measure of treatment response.

Nonetheless, this study derived a simple algorithm for the efficacy of rituximab in SSc-ILD using data from a randomized controlled trial with few missing data. Patients with even slight elevations of serum CRP would be good candidates for rituximab therapy in SSc-ILD.

## Supplementary material

Supplementary material is available at *Rheumatology* online.

## Supplementary Material

keae716_Supplementary_Data

## Data Availability

The data of this study are available from the corresponding author upon reasonable request.
