# Supplementary material for: Predictors of rituximab efficacy in systemic sclerosis-associated interstitial lung disease: machine-learning analysis of the DESIRES trial
Source: Rheumatology (Oxford). 2024 Dec 24;64(SI):SI114–21. doi: 10.1093/rheumatology/keae716 (PMC12695043; doi:10.1093/rheumatology/keae716)
Supplement: keae716_Supplementary_Data [file keae716_supplementary_data.zip › 22ac0_rhe-24-2452-File003.docx]

**Supplementary Information**

**Predictors of rituximab efficacy in systemic sclerosis-associated interstitial lung disease: machine-learning analysis of the DESIRES trial**

Ai Kuzumi^1†^, Koji Oba^2†^, Satoshi Ebata^1†^, Kosuke Kashiwabara^3^, Keiko Ueda^3^, Yukari Uemura^3,4^, Takeyuki Watadani^5^, Takemichi Fukasawa^1,6^, Shunsuke Miura^1^, Asako Yoshizaki-Ogawa^1^, Hidenori Kage^7^, Shinichi Sato^1^, Ayumi Yoshizaki^1,6*^

^1^ Department of Dermatology, Graduate School of Medicine, The University of Tokyo, Tokyo, Japan.

^2^ Department of Biostatistics, School of Public Health, The University of Tokyo, Tokyo, Japan.

^3^ Clinical Research Support Center, Tokyo University Hospital, Tokyo, Japan.

^4^ Biostatistics Section, Department of Data Science, Center for Clinical Sciences, National Center for Global Health and Medicine, Tokyo, Japan.

^5^ Department of Diagnostic Radiology, Graduate School of Medicine, The University of Tokyo, Tokyo, Japan.

^6^ Department of Clinical Cannabinoid Research, Graduate School of Medicine, The University of Tokyo, Tokyo, Japan.

^7^ Department of Respiratory Medicine, Graduate School of Medicine, The University of Tokyo, Tokyo, Japan.

^†^ These authors contributed equally to this work.

**^*^Correspondence and reprint requests to:**

Ayumi Yoshizaki, M.D., Ph.D., Department of Dermatology and Department of Clinical Cannabinoid Research, Graduate School of Medicine, The University of Tokyo; 7-3-1 Hongo, Bunkyo-ku, Tokyo 113-8655, Japan.

Telephone: +81-3-5800-8661, Fax: +81-3-3814-1503

**
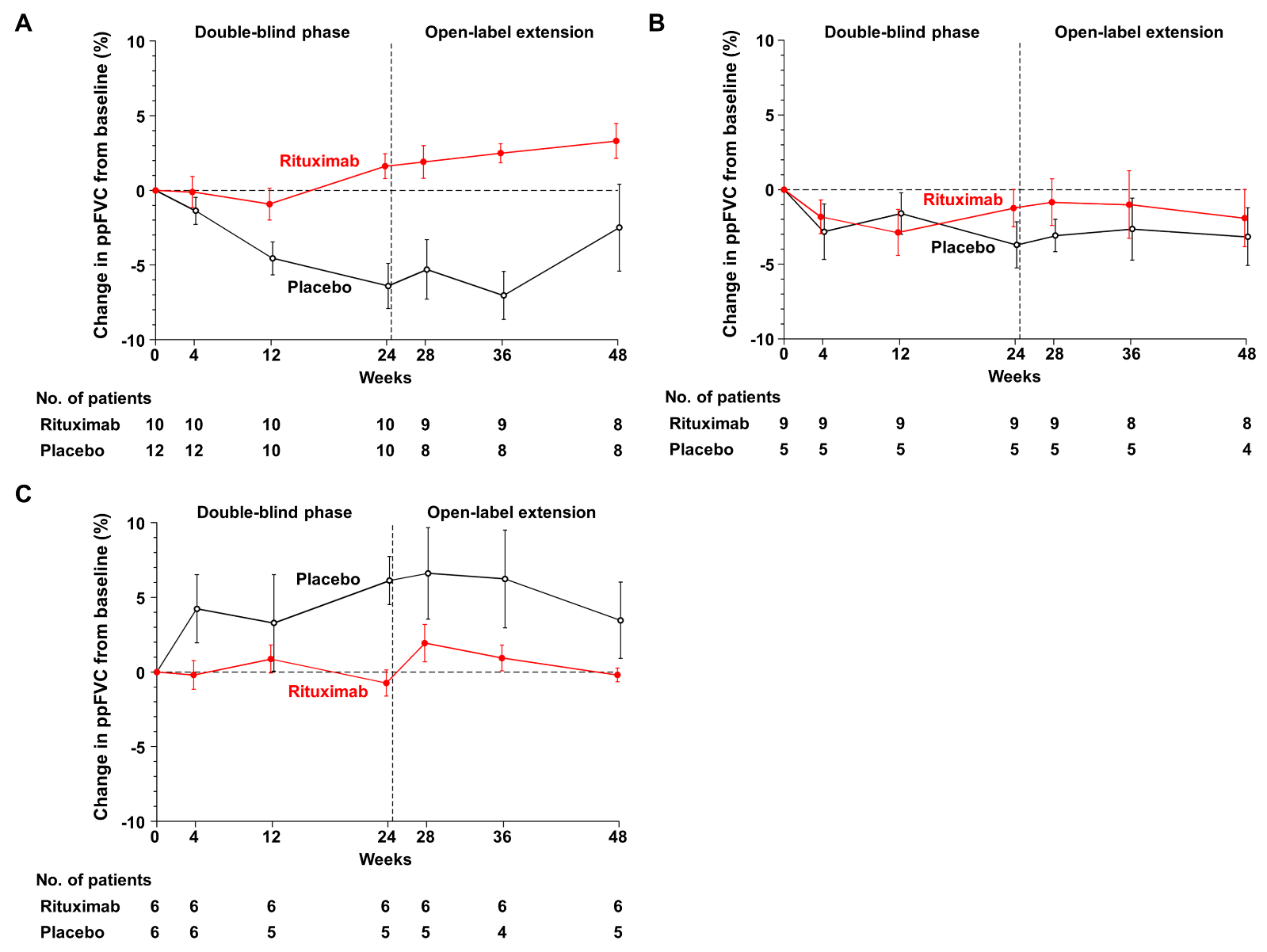
**

**Supplementary Figure S1.** **Change in ppFVC from baseline to 48 weeks in each subpopulation**

The mean ΔppFVC from baseline to 48 weeks in the placebo and rituximab groups in the following 3 subpopulations derived by the causal tree algorithm; (A) CRP levels ≥ 0.055 mg/dl (Leaf 1), (B) serum CRP levels < 0.055 mg/dl and serum KL-6 levels ≥ 364 U/ml (Leaf 2), and (C) serum CRP levels < 0.055 mg/dl and serum KL-6 levels < 364 U/ml (Leaf 3). Bars indicate the 95% CIs. CRP: C-reactive protein; FVC: forced vital capacity; KL-6: Krebs von den Lungen-6.
